# Dual-Energy Computed Tomography Imaging in Early-Stage Hepatocellular Carcinoma: A Preliminary Study

**DOI:** 10.1155/2022/2146343

**Published:** 2022-01-04

**Authors:** Jinping Li, Sheng Zhao, Zaisheng Ling, Daqing Li, Guangsheng Jia, Chenglei Zhao, Xue Lin, Yanmei Dai, Huijie Jiang, Song Wang

**Affiliations:** ^1^Department of Radiology, Second Affiliated Hospital of Harbin Medical University, Harbin 150081, China; ^2^Department of Radiology, Longhua Hospital Shanghai University of Traditional Chinese Medicine, Shanghai 200032, China

## Abstract

**Background:**

This study aims to evaluate the application of dual-energy computed tomography (DECT) for multiparameter quantitative measurement in early-stage hepatocellular carcinoma (HCC).

**Methods:**

The study retrospectively enrolled 30 patients with early-stage HCC and 43 patients with early-stage HCC who received radiofrequency ablation (RFA) and underwent abdomen enhanced CT scans in GSI mode. The GSI viewer was used for image display and data analysis. The regions of interest (ROIs) were delineated in the arterial phase and the venous phase. The optimal single energy value, CT values on different energy levels (40 keV, 70 keV, 100 keV, and 140 keV), the optimal energy level, the slope of the spectral attenuation curve, the effective atomic number (*Z*_eff_), iodine concentration (IC), water concentration (WC), normalized iodine concentration (NIC), and normalized water concentration (NWC) are measured and quantitatively analyzed.

**Results:**

The CT values of early-stage HCC at different single energy levels in dual phases were significantly different, and the single energy values were negatively correlated with the CT values. In the arterial phase and the venous phase, the optimal energy values for the best contrast-to-noise ratio were (68.34 ± 3.20) keV and (70.14 ± 2.01) keV, respectively. The slope of the spectral attenuation curve showed a downward trend at 40 keV, 70 keV, 100 keV, and 140 keV, but there was no statistically significant difference (*P* > 0.05). *Z*_eff_ was positively correlated with IC and standardized IC, but has no significant correlation with WC and NWC in dual phases.

**Conclusion:**

DECT imaging contains multiparameter information and has different application values for early-stage HCC, and it is necessary to select the parameters reasonably for personalized and comprehensive evaluation.

## 1. Introduction

Liver cancer is still one of the most malignant tumors that endanger human health in the world. According to the WHO statistics, the new cases of liver cancer will exceed 1 million by 2025 [1], and global cancer burden data of 2020 showed that liver cancer remains the sixth most common cancer and the third cause of cancer deaths worldwide [2], and the 5-year relative survival rate of liver cancer patients was only 18% [3]. Primary hepatocellular carcinoma (HCC) is the main pathotype of liver cancer, with insidious onset and prolonged course for many years, and most patients with HCC have chronic hepatitis and cirrhotic background. Early-stage HCC patients are always asymptomatic and have negative signs, and early-stage HCC is difficult to be detected by imaging on cirrhotic background [4].

In recent years, with the development of imaging technologies such as CT, MRI, and PET/CT, the early detection rate of HCC is gradually improving, but the early diagnosis of small HCC is still very challenging for radiologists. Some diagnostic imaging protocols for HCC, such as Liver Imaging Reporting and Data System (LI-RADS), provided a pipeline and summarized the standardized imaging features for HCC diagnosis. A pooled study pointed out that LI-RADS for CT/MRI was used to diagnose HCC with a sensitivity of 67% and a specificity of 93% [5]; in contrast, serum *a*-fetoprotein (AFP) is the most common screening method for HCC, with a specificity of 72%–90%, but a sensitivity of only 9%–32% [6]. In addition, only 70% of HCC patients showed abnormally elevated serum AFP, and about 30% of patients were AFP negative [7]. This result implied that imaging methods have an advantage in diagnostic sensitivity and specificity compared to laboratory tests, but some lesions still failed to meet the typical imaging criteria, and it was crucial to effectively differentiate typical HCC by imaging. Meanwhile, due to the subjectivity of radiologists in diagnosing HCC, there is often variation in the results of different readers, which directly leads to a strong dependence of imaging evaluation on the experience and expertise of the readers [4].

X-rays will form a characteristic absorption curve during the attenuation process of penetrating materials [8], and the materials' absorption attenuation coefficient of X-rays varies with the X-ray energy. In general, conventional liver CT scans are performed by a single energy X-ray, and different elements produce the same or similar Hounsfield unit at a single energy, which leads to polychromatic data [9]. In contrast, DECT can acquire two sets of images of the same tissue using different photon spectra (high and low kVp) [10], and thus, by adjusting the photon spectrum, the optimal single energy with optimized contrast-to-noise ratio (CNR) can be obtained, which in turn improves the detection rate of smaller tissue density differences as well as small lesions.

Summarily, this retrospective study aimed at exploring the role of multiparameter obtained by DECT imaging in the quantitative measurement of HCC.

## 2. Methods

### 2.1. Patients Population

We retrospectively collected cases diagnosed with early-stage HCC by DECT from October 2018 to January 2021 in the Second Hospital of Harbin Medical University, and all cases were confirmed by surgical pathology. This study was approved by the Ethics Committee of the Second Hospital of Harbin Medical University, and all participants signed informed consent forms for the application of iodine contrast agent.

Finally, 30 patients were enrolled and the exclusion criteria were as follows: (1) severe impairment of cardiac, hepatic, renal function, or hematopoietic system; (2) receiving preoperative antitumor therapy, such as radiotherapy or targeted therapy; (3) portal hypertension, portal vein, or intrahepatic vein thrombosis; and (4) peritoneal fluid excess.

Additionally, from October 2018 to January 2021, we collected 43 patients who confirmed early-stage HCC by imaging diagnosis and met the indications of radiofrequency therapy in our hospital. All patients did not receive other pre-RFA antitumor therapy.

### 2.2. CT Protocol

Gemstone Energy Spectrum CT (Discovery CT750 HD) was used to perform the scan. Informed consent was obtained and signed by patients, and the patients fasted 4–6 hours before CT scanning, the entire liver was scanned in the preset abdominal LIVER + GSI mode, and arterial phase (AP), portal vein phase (PVP), and delayed phase were routinely acquired. The arterial phase was determined using automated scan triggering software (Smart Prep, GE Healthcare). The CT value of abdominal aorta at the level of the celiac artery was monitored to trigger the scan with a monitoring threshold of 150 HU and a 6-second delay in starting the scan after reaching the threshold. Venous phase and delayed phase were obtained at 30 seconds and 180 seconds, respectively, after the arterial phase with the same range of the plain scan. A double-barrel high-pressure syringe (Oelrich, Germany) was used to establish venous access for drug administration in the anterior elbow vein at a rate of 3 mL/s, and 60–80 mL of nonionic iodine contrast agent (Iobitridol, Guerbet, France) was injected, and 30 mL of saline was injected at the same rate after the drug injection was completed. The tube current varies slightly from patient to patient, with a range of about 200–400 mA and a voltage of 100–120 kV (80 kVp–140 kVp instantaneous high-speed switching). Layer thickness was 5.0 mm, layer spacing was 5.0 mm, pitch was 0.984 : 1, and reconstruction interval was 1.25 mm.

### 2.3. Radiofrequency Ablation Procedure

According to the location of HCC found by pre-FRA imaging, appropriate body positions were selected, and the puncture point and needle route were designed. After routine disinfection, towel laying, the skin of the puncture site was incised about 2 cm under local anesthesia with 2% lidocaine, percutaneous transhepatic radiofrequency ablation was performed under ultrasound guidance, and the radiofrequency needle reached a depth of about 0.5–1 cm from the subcutaneous to the inner abdominal wall, and then we asked the patient to hold his/her breath, reconfirmed the puncture path on ultrasound images, and inserted the needle quickly. After laying the needle, we firstly confirmed that the electrode needle is in the exact position of the setting and that the tip of the ablation needle exceeds the distal edge of the tumor by about 0.5 cm, after which ablation is performed. For lesions with clear tumor donor vessels, ablation was first performed by puncturing through normal liver tissue to the tumor donor vessels. Then, we activated the power and performed ablation according to the preset treatment plan and observed the ablation process in real time. Hot-tip cautery was performed by cauterizing the needle tract and withdrawing the ablation needle at the end of treatment.

### 2.4. Data Processing and Analysis

The DECT data were transferred to a postprocessing workstation (AW4.6, GE, USA) for postprocessing and analysis using the DECT Viewer software package. Two radiologists with more than 10 years' experience in abdominal imaging diagnosis performed data measurements and image analysis. Firstly, the optimal energy value for the optimal CNR curve was obtained, and then the data were measured at this energy level. The radiologists selected the largest dimension of the lesion diameter in the cross section and manually outlined regions of interest (ROIs), necrotic areas, and blood vessels within the HCC lesions were avoided. In patients receiving RFA, the ROIs should be placed as close to the edge of the lesion as possible and obvious ablation zone was avoided. The area of the ROIs was approximately 1/3 of the lesion, and each ROI was triple measured and averaged. The measured data in this study included the following: (1) CT values of HCC lesions at 40 keV, 70 keV, 100 keV, and 140 keV; (2) the slope of spectral attenuation curves (*λ*) at different energy intervals, including 40–70 keV, 40–100 keV, and 40–140 keV, was calculated by the energy spectrum curve slope formula, *λ*_1 − 2_ = (CT_1_ − CT_2_)/(keV_2_ − keV_1_); (3) iodine concentration (IC), water concentration (WC), and effective atomic number (*Z*_eff_) were obtained in the AP and PVP, and the difference between IC and WC in the AP and PVP was calculated and expressed as ΔPVP − AP; (4) normalized iodine concentration (NIC) and normalized water concentration (NWC) were calculated as follows: NIC = IC_lesion_/IC_aorta_, NWC = WC_lesion_/WC_aorta_; and (5) *Z*_eff_, IC, NIC, and the slope of the spectral attenuation curve at 40–100 keV interval were measured at baseline (before RFA) and 1 year after RFA.

### 2.5. Statistical Analysis

Statistical analysis was performed using SPSS version 19.0 and GraphPad Prism version 7.00 for Windows. All data were expressed as mean ± SD. The paired *t*-test was performed to compare CT values of HCC lesions on different energy levels and the slopes of spectral attenuation curves at different energy intervals. Pearson's correlation was used to analyze the relationship between *Z*_eff_ and IC, WC, NIC, and NWC; the closer the absolute value of Pearson's correlation coefficient (*r*) is to 1, the higher the correlation of these two variables is recognized. A two-sample *t*-test was used to compare the spectral parameter before RFA and 1 year after RFA for early-stage HCC, and *P* < 0.05 was considered a statistically significant difference.

## 3. Results

### 3.1. Patients

We collected 30 patients with early-stage HCC diagnosed by Gemstone Energy Spectral CT imaging from October 2018 to January 2021, and all patients had solitary lesions. Of these patients, 21 were males aged 36 to 63 years and 9 were females aged 50 to 66 years. A total of 25 cases (83.33%) had liver cirrhosis background, and a summary of patient demographics and clinical characteristics is shown in Table 1.

Additionally, we enrolled 43 patients (31 males, aged 30–53 years; 12 females, aged 40–60 years) with early-stage HCC who received ultrasound-guided RFA, and all of them had solitary lesions.

### 3.2. CT Values of Early-Stage HCC at Different Single Energy Levels and the Best CNR

**Figure 1:**
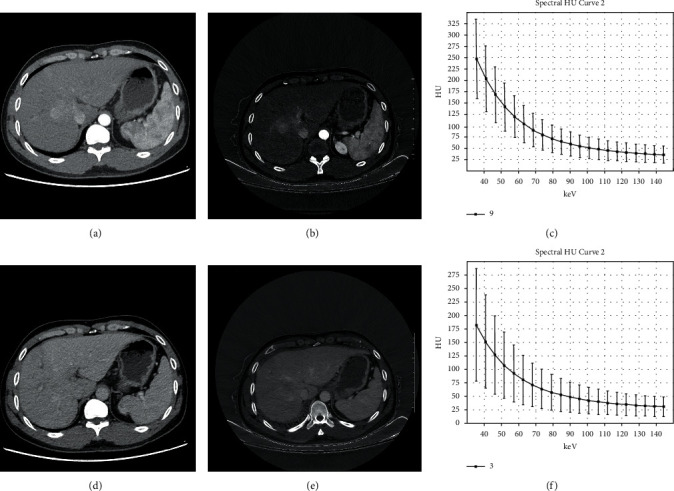
The CT values of hepatocellular carcinoma lesions decreased at different energy levels. (a) The arterial phase image at 70 keV. (b) The arterial phase iodine-based image at 70 keV. (c) Spectral curve of lesion on arterial phase. (d) The portal venous phase image at 70 keV. (e) The portal venous phase iodine-based image at 70 keV. (f) Spectral curve of lesion on portal venous phase.

The comparison of CT values of early-stage HCC at different energy levels in the AP and PVP is shown in Table 2. The CT values of early-stage HCC at different single energy levels in dual phases were different, and the single energy values were negatively correlated with the CT values, the general trend was that the higher the single energy values, the lower the CT values. At low energy levels, the CT values decreased more obviously. At 70 keV and 100 keV, the CT values were statistically different in the AP and PVP (*P* < 0.05). The optimal single energy values in the AP and PVP were (68.34 ± 3.20) keV and (70.14 ± 2.01) keV, respectively (Figure 1).

### 3.3. Spectral Parameter of Early-Stage HCC

At different energy intervals (40–70 keV, 40–100 keV, and 40–140 keV), there was no significant difference in the slope of the spectral attenuation curve between AP and PVP (*P* > 0.05). However, at these three energy intervals, the slope of the spectral attenuation curve showed a downward trend in the AP and PVP. Further, at low energy levels, the slope of the spectral attenuation curve significantly decreases (Table 3), but there is no statistically significant difference (*P* > 0.05). IC, WC, NIC, and NWC in the AP and PVP are shown in Table 4, IC was slightly higher in the AP than in the PVP, while WC and NWC in the PVP were slightly higher than in the AP, and NIC was higher in the PVP than in the AP, ΔPVP-AP of NIC was larger than that of IC, WC, and NWC.

**Figure 2:**
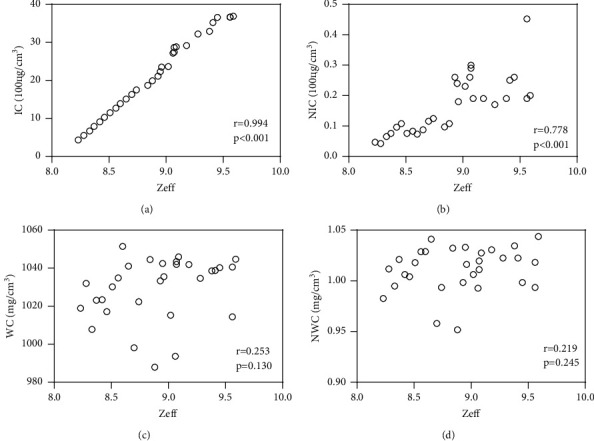
Arterial phase spectral parameter. (a) The relationship between *Z*_eff_ and IC on arterial phase. (b) The relationship between *Z*_eff_ and N IC on arterial phase. (c) The relationship between *Z*_eff_ and WC on arterial phase. (d) The relationship between *Z*_eff_ and NWC on arterial phase.

**Figure 3:**
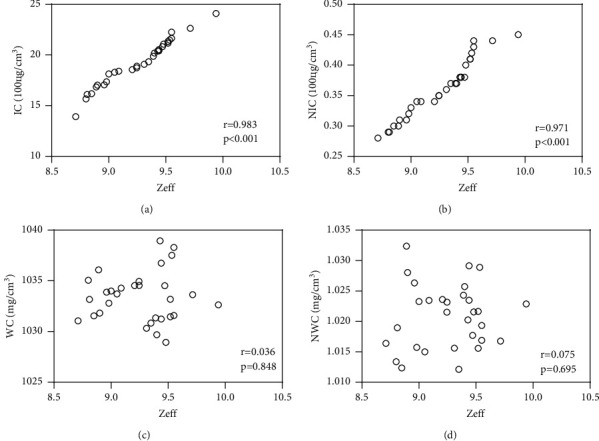
Portal venous phase spectral parameter. (a) The relationship between *Z*_eff_ and IC on portal venous phase. (b) The relationship between *Z*_eff_ and NIC on portal venous phase. (c) The relationship between *Z*_eff_ and WC on portal venous phase. (d) The relationship between *Z*_eff_ and NWC on portal venous phase.

Both IC and NIC exhibited a significant positive correlation with *Z*_eff_ in the AP and PVP (*P* < 0.001), with correlation coefficients of 0.994 and 0.778 in the AP, and 0.983 and 0.971 in the PVP, respectively. Conversely, both WC and NWC were not significantly correlated with *Z*_eff_ in the AP and PVP. In the AP, the correlation coefficients were 0.253 (*P*=0.130) and 0.219 (*P*=0.245), respectively (Figure 2). And in the PVP, the correlation coefficients were 0.036 (*P*=0.848) and 0.075 (*P*=0.695), respectively (Figure 3).

### 3.4. Pre-RFA and Post-RFA Iodine Concentration-Related Spectral Parameter in the Arterial Phase

**Figure 4:**
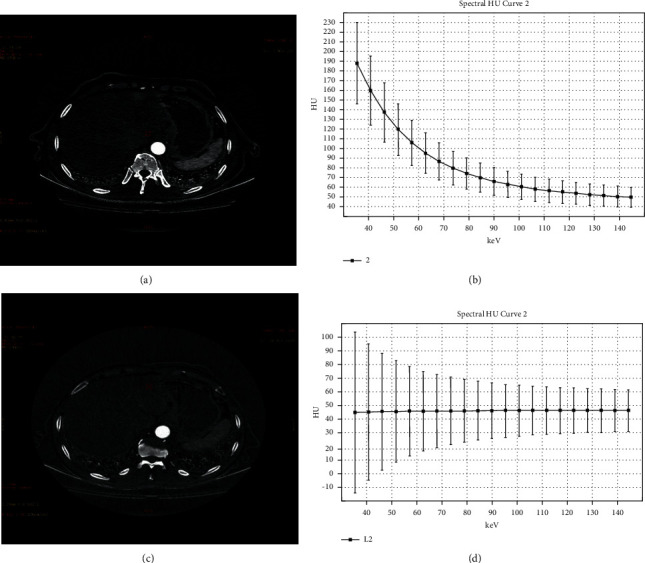
The pre- and post-RFA iodine-based images on arterial phase and corresponding slope of the spectral attenuation curves. (a) The iodine-based image before RFA. (b) The slope of the spectral attenuation curve before RFA. (c) The iodine-based image after RFA. (d) The slope of the spectral attenuation curve after RFA.

*Z*_eff_, IC, NIC, and *λ*40-100 keV were statistically different before RFA and 1 year after RFA at 70 keV (*P* < 0.05), and pre-RFA parameters were lower than post-RFA parameters in *Z*_eff_, IC, NIC, and *λ*40-100 keV, and the decrease of IC and NIC values was more significant. The slope of the spectral attenuation curve after RFA was flat and close to a straight line (Figures 4(a) and 4(b)), and the scatter plots before and after RFA were mostly distributed at different intervals, mostly concentrated in the range of 70–110HU before RFA and in the range of 20–70HU after RFA, with a more significant decrease (Figures 4(c) and 4(d)).

## 4. Discussion

DECT is a new technology; unlike conventional CT, fast kilovoltage-switching techniques for DECT obtain many parameters for energy spectrum analysis and decompose materials for a qualitative study of tissues [11]. Different levels of monochromatic energy spectrum can meet the diagnostic needs of different diseases, and different energy spectral parameters have different applications for disease diagnosis and efficacy assessment [12]. Moreover, DECT has irreplaceable advantages in reducing X-ray sclerosis artifacts and CNR, optimized image quality and enhanced the display of hypovascular lesions, and benefited radiologists for disease diagnosis and lesion detection [4]. Studies have shown that tissue contrast increases significantly at low energy values; especially for hypervascular lesions, the enhanced contrast with surrounding normal tissues was vital, increasing the visibility of early-stage HCC lesions that were difficult to find in conventional enhanced CT [13, 14].

Secondly, image quality is critical for morphological evaluation, and the most common method for evaluating image quality is CNR. Studies had indicated that, compared with conventional CT, monochromatic energy images at 70 keV have the lowest noise and highest CNR, which can improve the CNR of abdominal organs by 13.8% to 24.7% [15, 16]. Therefore, the energy images with optimized noise and contrast between tissues can be obtained by adjusting kiloelectron volts, thereby increasing the density difference between tissues. At present, low-energy keV monochromatic energy images are mainly used to detect iso-attenuation or smaller lesions in the abdominal parenchymal organs [8]. In this study, the image quality has not been specifically scored, and optimal single energy values automatically obtained by the DECT Viewer software were (68.34 ± 3.20) keV and (70.14 ± 2.01) keV in AP and PVP, respectively. There is no significant difference between AP and PVP; both were around 70 keV. Meanwhile, Lv et al. demonstrated that monochromatic energy images at low-energy keV (40–70 keV) can improve the detection rate of small liver nodules without affecting the overall quality, which is basically consistent with our conclusion [15].

Thirdly, we compared the CT values on different energy levels to determine the relatively accurate energy level for accurate CT values, and the purpose was to provide accurate measurements of lesions. This study compared and analyzed the CT values of lesions at 40 keV, 70 keV, 100 keV, and 140 keV in dual phases. The results showed that the CT values measured at 40 keV and 140 keV were not statistically significant. These two energy levels are close to the lowest and highest single energy level, respectively. X-ray attenuation is obvious on the lowest single energy level, which results in amplified measured CT values and low CNR. Conversely, the highest single energy level is closer to polychromatic X-ray, which causes beam hardening and lowers the degree of arterial enhancement CNR [17, 18]. Further, we observed that the difference between the CT values at 70 keV and 100 keV was statistically significant, so the CT values measured at the optimal single energy or 70 keV were relatively more accurate and more accurately reflected the true degree of lesions enhancement.

The spectral attenuation curve shows the attenuation of materials on different energy levels, which is mainly determined by the molecular structure of materials. In this study, the spectral attenuation curves in all HCC lesions were descending. The curve was steep at the low energy level and was flat at the high energy level. In order to express the meaning of the slope of the spectral attenuation curve more accurately, this study divided the spectral attenuation curves into three different energy intervals, namely, 40–70 keV, 40–100 keV, and 40–140 keV. The results showed that the slopes of the three energy interval curves gradually decreased. Although the slope of the energy spectrum curves at the three energy intervals is not statistically different, it may be related to the arterial blood supply of lesions and the clearance rate of contrast agent. However, the slope of spectral attenuation curves in AP was superior to that in PVP, which is consistent with the arterial blood supply of HCC lesions. Significant enhancement and higher iodine content in lesions lead to more attenuation of X-rays, so the slope of the curve is steeper, and the results indicated that 40–100 keV energy intervals are reliable and effective for data collection and analysis. In addition, as a new quantitative parameter, *Z*_eff_ is an important method for accurate analysis of inorganic materials in DECT, can directly reflect the effective atomic number of the materials in ROIs, and then qualitatively determine the materials [19]. Previous study has concluded that *Z*_eff_ can be used to identify different materials, thereby indirectly reflecting the internal structural characteristics of the tissue [20]. We found that *Z*_eff_ has a positive correlation with IC and NIC in dual phases, and conversely, there is no correlation between WC, NWC, and *Z*_eff_ in both AP and PVP. This result suggested that *Z*_eff_ can be used as a quantitative parameter to differentiate materials. Furthermore, compared with conventional enhanced images, iodine concentration maps are more accurate and reliable for CT value measurement [21]. In other words, the IC is more accurate than the CT value in assessing the blood supply of the lesion.

Finally, we conducted a preliminary study to evaluate the tumor response of HCC lesions by DECT after RFA. CT was a common imaging follow-up method for post-RFA HCC patients, and conventional enhanced CT was used to judge the efficacy of RFA according to the degree of lesions enhancement in the past. However, with the emergence of DECT, many parameters that evaluate the efficacy of RFA in multiple directions have unique advantages and promote the clinical application value of CT to a new height. Compared with and pre-RFA lesions, we found that Z_eff_, IC, NIC, and *λ*40-100 keV showed a significant decrease in post-RFA lesions, and the difference was statistically significant, IC and NIC decreased significantly, pre-RFA and post-RFA were (21.59 ± 10.43) 100 *μ*g/cm^3^ and (2.77 ± 1.98) 100 *μ*g/cm^3^, 0.17 ± 0.09 and 0.02 ± 0.01, and Z_eff_ decreased from 8.90 ± 0.41 to 7.62 ± 0.39; the result indicated that the arterial blood supply of the lesion appeared to be significantly decreased after RFA and the treatment effect was remarkable, which implied that the post-RFA lesions lack blood supply and have no signs of recurrence or residual.

Furthermore, the multiparameter obtained by DECT has achieved many applications in the management of HCC. Kaltenbach et al. found that normalized iodine uptake can effectively differentiate net liver metastases from HCC in noncirrhotic livers with a sensitivity and specificity of 100% and 90.2%, respectively [22], and Lv et al. found that quantitative image analysis using DECT could improve the sensitivity for differentiating between small hepatic hemangiomas and small hepatocellular carcinoma lesions [12]. For HCC patients after TACE, Yue et al. dictated that *λ* showed excellent performance in distinguishing tumor necrotic areas from tumor active areas. On the other hand, the combination of DECT with radiomics and deep learning algorithms may be a new direction in the future. Homayounieh et al. demonstrated that quantitative iodine and radiomic features of lesion margins can effectively differentiate benign from malignant liver lesions [23]. Although there are few similar studies, we believe that further studies may build a more robust model based on DECT, which brings us a more accurate and feasible diagnostic path that can exclude the interference of subjective factors of radiologists and yield more accurate diagnostic results.

There are some limitations in this study. First of all, the size and pathological differentiation of HCC are not discussed. Different sizes and pathological differentiation will affect the measurement of energy spectral parameters and the prediction of each parameter on the diagnostic efficacy of HCC. Secondly, sample size is slightly small, and it is necessary to conduct group research and discussion with large sample size and multiple clinical and pathological factors.

In summary, this study performed DECT imaging for early-stage HCC, analyzed the diagnosis value of imaging parameters, and applied DECT imaging to evaluate post-RFA HCC lesions. DECT imaging contains multiparameter information and has different application values for early-stage HCC, and it is necessary to select the parameters reasonably for personalized and comprehensive evaluation.

## Figures and Tables

**Table 1:** Characteristics of patients.

	Male (*N* = 21)	Female (*N* = 9)	*P* value
Age^a^	53(46,60)	58(57,62)	0.060
Etiology^b^			
HBV	11 (52.38%)	5 (55.56%)	1.000
Others	10 (47.62%)	4 (44.44%)	
AFP (ng/mL)^b^			
≤9	12 (57.14%)	2 (22.22%)	0.118
>9	9 (42.86%)	7 (77.78%)	
CEA (ng/mL)^b^			
≤5	11 (52.38%)	4 (44.44%)	1.000
>5	10 (47.62%)	5 (55.56%)	
BMI (kg/m^2^)^b^			
<18.5	3 (14.29%)	3 (33.33%)	0.345
18.5–24.9	10 (47.62%)	2 (22.22%)	
>25	8 (38.10%)	4 (44.44%)	

^a^Median (IQR); ^b^*n*(%).

**Table 2:** CT values of early-stage hepatocellular carcinoma on different energy levels.

Scan phase (keV)	AP	PVP	*t*	*P*-value
40	232.55 ± 45.74	190.42 ± 80.18	2.001	0.055
70	102.00 ± 17.42	82.79 ± 22.18	2.998	0.005
100	66.25 ± 10.21	56.68 ± 15.45	2.269	0.030
140	52.50 ± 9.53	47.53 ± 23.97	0.843	0.407

*P* < 0.05 represents a statistically significant difference.

**Table 3:** The slope of the spectral attenuation curves on dual phases at different energy intervals (HU/keV).

Scan phase	AP	PVP	*t*	*P* value
*λ*(40–70 keV)	4.39 ± 1.08	3.89 ± 1.61	1.141	0.262
*λ*(40–100 keV)	2.79 ± 0.70	2.44 ± 1.00	1.278	0.209
*λ*(40–140 keV)	1.82 ± 0.45	1.58 ± 0.64	1.316	0.197

*P* < 0.05 represents a statistically significant difference.

**Table 4:** The IC, WC, NIC, and NWC on dual phases.

Scan phase (*n* = 30)	AP	ΔPVP − AP	PVP
IC (100 *μ*g/cm^3^)	21.59 ± 10.43	2.34 ± 8.18	19.25 ± 2.33
WC (mg/cm^3^)	1,029.25 ± 16.43	−4.15 ± 14.23	1,033.41 ± 2.48
NIC	0.17 ± 0.09	−0.19 ± 0.05	0.36 ± 0.05
NWC	1.01 ± 0.02	−0.01 ± 0.02	1.02 ± 0.01

## Data Availability

The datasets used and/or analyzed during the current study are available from the corresponding author on reasonable request.
